# 

**Published:** 1828-05

**Authors:** 


### MONTHLY LIST OF MEDICAL BOOKS.

*[Medical Works cannot be entered on this List except a copy be sent for the purpose; the titles of Books having frequently been transmitted to us, as published, which have not appeared for weeks, or even months, after.]*

Transactions of the Association of the Fellows and Licentiates of the King and Queen's College of Physicians in Ireland. Volume V.—8vo. pp. 576. Cumming, Dublin, 1828.

Surgical Observations on the Treatment of Chronic Inflammation in various Structures; particularly as exemplified in the Diseases of the Joints. By JOHN SCOTT, Surgeon to the London Ophthalmic Infirmary, and assistant Surgeon to the London Hospital.—8vo. pp. 291. Longman and Co. London, 1828.

Observations on Fever. By R. WADE, Member of the Royal College of Surgeons. Second edition.—8vo. pp. 83. Burgess and Hill, London, 1828.

A Treatise on Gout, Apoplexy, Paralysis, and Disorders of the Nervous System. By A. Rennie, Surgeon, &c.—8vo. pp. 213. Burgess and Hill, London, 1828.

Hints to Young Medical Officers of the Army on the Examination of Recruits, and respecting the feigned Disabilities of Soldiers; with Official Documents, and the Regulations for the Inspection of Conscripts for the French and Prussian Armies. By Henry Marshall, Surgeon to the Forces. —8vo. pp. 224. Burgess and Hill, London, 1828.

Medical Botany, No. XIII.; containing plates of the Inula Helenium, Ricinus communis, Althæa officinalis, and Strychnos Nux Vomica. No. XIV. containing Fraxinus Ornus, Valeriana officinalis, Delphinum Staphisagria, and Daucus Carota. No. XV. Punica Granatum, Santonica Absinthium, Carum Carui, and Convolvulus Scammonia. No. XVI. Linum Usitatissimum and Catharticum, Cephaelis Ipecacuanha, Oxalis Acitosella, and Bryonia Diorca.

*** The plates continue to be executed in the same masterly manner as in the earlier Numbers.

